# The B-Side of Cancer Immunity: The Underrated Tune

**DOI:** 10.3390/cells8050449

**Published:** 2019-05-13

**Authors:** Anne Largeot, Giulia Pagano, Susanne Gonder, Etienne Moussay, Jerome Paggetti

**Affiliations:** Tumor Stroma Interactions, Department of Oncology, Luxembourg Institute of Health, L-1526 Luxenbourg, Luxembourg; anne.largeot@lih.lu (A.L.); giulia.pagano@lih.lu (G.P.); susanne.gonder@lih.lu (S.G.)

**Keywords:** B lymphocytes, tumor microenvironment, immunotherapy, tumor immunity, Breg

## Abstract

Tumor-infiltrating lymphocytes are known to be critical in controlling tumor progression. While the role of T lymphocytes has been extensively studied, the function of B cells in this context is still ill-defined. In this review, we propose to explore the role of B cells in tumor immunity. First of all we define their dual role in promoting and inhibiting cancer progression depending on their phenotype. To continue, we describe the influence of different tumor microenvironment factors such as hypoxia on B cells functions and differentiation. Finally, the role of B cells in response to therapy and as potential target is examined. In accordance with the importance of B cells in immuno-oncology, we conclude that more studies are required to throw light on the precise role of B cells in the tumor microenvironment in order to have a better understanding of their functions, and to design new strategies that efficiently target these cells by immunotherapy.

## 1. Introduction

Immunity is a complex and finely regulated process which involves the coordinated action of different cell types. B cells (Bursal–derived lymphocytes) are the central players of the humoral immunity through their capacity of immunoglobulins (antibodies) production. In first instance, antigens are recognized by the B cell receptor (BCR) composed of membrane anchored-immunoglobulin and co-receptor molecules. Upon first antigen encounter, naïve mature B cells are turned into activated B cells, capable of proliferation and differentiation into plasma cells (PCs), which produce and release antibodies [1]. Mature B cells are divided into three main subsets: B1 B cells, mainly found in peritoneal and pleural cavities; B2 or follicular (FO) B cells, which are the most abundant and are located in the B cell areas of lymph nodes, Peyer’s patches and spleen; and marginal zone (MZ) B cells, sitting in the marginal sinus of the spleen [2]. B cells of different subsets vary in terms of their location and in the way they are activated in a T-dependent or a T-independent way. Polysaccharides or lipidic antigens mostly cause a T-independent response, which leads to the production of short-lived plasma cells. This is the case of B1 and MZ B cells that mostly bind non-proteic antigens. On the other side, the T-dependent response executed by FO B cells (and in some cases by MZ B cells), provides for the presentation of the antigen to follicular T helper cells (Tfh) through MHC class II molecules, which in turn through CD40, IL-21 and IL-4 production, stimulate B cell activation and maturation into short-lived plasma cells [3]. FO B cells can also undergo further maturation in the germinal centers (GCs) where they become either long-lived plasma cells or memory B cells. Contrary to MZ and B-1 B cells, FO B cells produce high affinity antibodies which are very specific to one antigen [4]. Interestingly, B cells play an important role in immunity independently of their antibody production function, particularly through secretion of cytokines which can affect T cells, dendritic cells (DC), lymphoid tissue reorganization and neogenesis. 

A poorly characterized but important B cell subset is represented by regulatory B cells (Bregs), functionally defined by their capacity to inhibit T cell mediated immunity. However, different types of Bregs have been depicted, arising from different B cell subpopulations, making their studies difficult. One of the major hallmark of this population is the production of inhibitory cytokines such as interleukin-10 (IL-10), IL-35 or transforming growth factor beta (TGF-β) [5,6,7]. For this review, we will consider Bregs as cells which can suppress immunity independently of their phenotype.

In the context of tumor immunity, T cells are widely studied and characterized. Indeed, they attracted the main attention for several reasons: infiltration of cytolytic T cells is associated with good prognosis [8] while regulatory T lymphocytes (Tregs) suppress anti-tumor response [9]. Moreover, the different T cell subpopulations have been well characterized during the last decades both functionally and phenotypically. Notably, current immunotherapies based on the immune checkpoint blockade are designed to target these cells and reactivate the anti-tumor immunity [10]. Despite the great advance that these new therapies represent, a certain number of patients do not benefit from them, and efforts should be made to better understand other immune players in this tumor battlefield in order to develop better alternatives. In some cancer types, B cells represent an important proportion of infiltrating cells, as shown in breast cancer [11,12,13], epithelial ovarian cancer [14], melanoma [15], non-small-cell lung carcinoma [16,17], renal cell carcinoma [18]. B cells can associate with T cells and organize in tertiary lymphoid structures (TLS) within the tumor, where it is believed that naïve T cells can be activated [19], which highlights the potential role of B cells in modulating anti-tumor immunity. In the majority of cancer types, the infiltration of B cells is associated with a good prognosis [20], as for T cells however, B cells are associated with different activities depending on their phenotype, which does not facilitate their study.

In this review we explore the dual role of B cells in tumor immunity, then depict the tumor microenvironment factors that influence their functions and finally examine the role of B cells in therapy.

## 2. Role of B Cells in Pro- and Anti-Tumor Immunity

**Figure 1:**
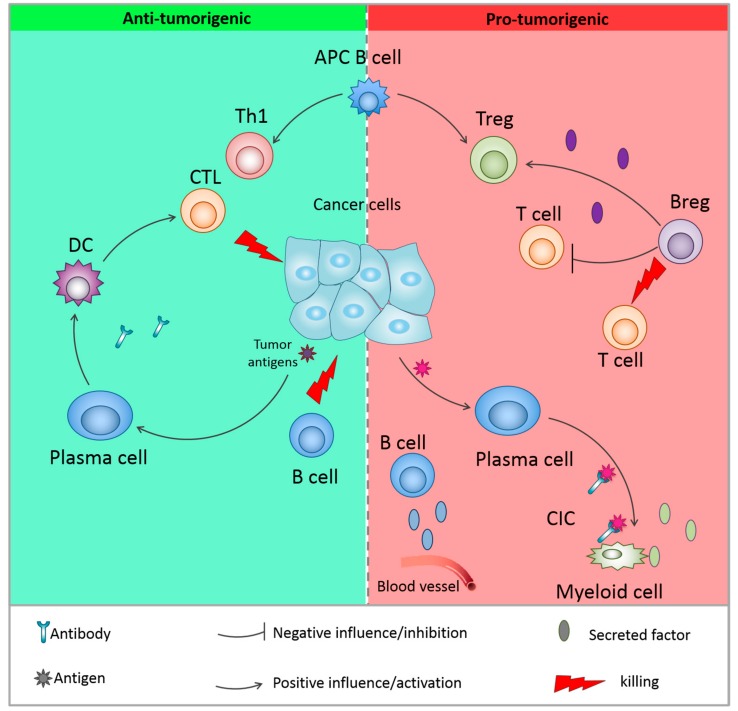
Dual role of B cells in the tumor microenvironment. B cells can have anti-tumor activities through the recognition of tumor-specific antigens and antibody production, antigen presenting cell (APC) function or direct killing of cancer cells. They can also be associated to pro-tumorigenic activities, through activation of myeloid-derived suppression cells (MDSC), production of pro-tumorigenic cytokines and activation of immunosuppressive regulatory T cells. The pro-tumoral activity is mainly mediated by regulatory B cells. DC = dendritic cells; CTL = cytotoxic T cells; Th1 = type 1 T helper cell; APC = antigen presenting cell; CIC: circulating immune complex.

Recently, the importance of B cells in the tumor microenvironment (TME) has been more and more investigated and discussed, which has led to controversial evidences in the field of tumor immunology. The role of B cells in the TME is diverse and besides the secretion of antibodies and cytokines, B cells are able to modulate T cell and innate immune responses and also, recognize antigens, regulate antigen processing and presentation [21]. The balance between B cell subtypes (which are characterized by the expression of specific markers) and their activities affects pro– or anti–tumorigenic function. In light of these contradictory activities, it is thus not surprising that discrepancies are observed when the prognostic value of B cells is studied. Indeed, B cells have been described as being markers of both good and bad prognosis [20]. Here, we will summarize the dual role of the heterogeneous B cell populations in pro- and anti-tumor immunity (Figure 1, Table 1).

### 2.1. Anti-Tumor Activity of B Cells

#### 2.1.1. Antibody Production

Tumor cells can trigger a humoral response due to the expression of specific antigens, which can be a consequence of mutations (neoantigens), the overexpression of genes, aberrant post-translational modification, the expression of a specific differentiation marker (for example CD20 in B related lymphoma/leukemia) or the expression of marker normally found in a restricted tissue, such as the Cancer–testis antigens, normally restricted to testis, which are found in melanoma and other tumors [32]. B cell-mediated antibodies production can lead to killing of tumor cells through the complement cascade activation, phagocytosis by macrophages and activation of tumor killing activity of NK [24]. In 2009, Reuschenbach and collaborators exploited published data from 145 articles in order to better describe the humoral response developed against tumor antigens. A majority of the antigens that have been studied were derived from overexpressed or mutated antigens. Nevertheless, as mentioned in this study, it is not clear if antibody production reflects a proper B cell response against the tumor, or is just an irrelevant consequence of exposure to the antigens [33]. However, several lines of experimental evidence tend to support the idea that antibody production could efficiently target and control tumor growth. In vitro–activated B cells obtained from tumor draining lymph nodes produce high quantity of antibodies when co-cultured with tumor cells, which can mediate complement–dependent specific killing of the tumor. When injected in tumor-bearing mice, these cells lead to a regression of the tumor [25]. The injection of tumor specific antibodies induces complement–dependent tumor regression in a model of large cell lung carcinoma [34]. More recently, Carmi et al., using an allogenic tumor rejection model (B16F10 melanoma cells, originated from C57BL/6 mice, injected in 129S1 mice), elegantly demonstrated that B cells-produced antibodies at the very early stage of tumor development activate dendritic cells (DC) which in turn trigger a cytotoxic T cell response to control tumor growth [35].

#### 2.1.2. Other Functions of B Cells

B cells have the capacity to directly kill tumor cells. Indeed, Tao and colleagues showed that CD19^+^ IL10^−^ B cells derived from tumor-draining lymph nodes (and in vitro activated) express FASL which triggers the apoptotic signal in 4T1 murine breast cancer cells expressing FAS [6]. This direct killing activity of CD19^+^ B cells is exacerbated by the production of IL-17A in the TME [36]. Another example of direct cytotoxicity is represented by CpG–activated B cells, which are able of killing cancer cells through TRAIL/Apo-2L–related pathway [26]. Moreover, upon IL-21 stimulation, B cells are capable of producing granzyme B. In chronic lymphocytic leukemia (CLL), IL-21–stimulated leukemic B cells can kill non stimulated ones [30].

In addition, specific B cell subtype expressing B220, CD19 and CD11c can act as antigen presenting cells (APCs) [37,38]. CD20^+^ B cells can be found in close proximity of T cells in several types of cancer, including when dendritic cells are not present, such as ovarian cancer, which suggest that they can play the role of APC in this case [14]. In non–small–cell lung cancer, infiltrating B cells can act as APC to CD4^+^ T cells. Interestingly, 2 classes of APCs were identified in this setting: activated (CD69^+^ HLA-DR^+^ CD27^+^ CD21^+^) or exhausted (CD69^+^ HLA-DR^+^ CD27^−^ CD21^−^), both displaying opposite functions. Whereas the activated B cell APCs were able to induce Th1 differentiation, exhausted ones led to the generation of Tregs [39]. Other B cell functions have been described such as secretion of cytokines, which can trigger an active T cell response. However, there is no evidence that they are associated with anti-tumor activity.

### 2.2. Pro-Tumor Activity of B Cells

#### 2.2.1. Conventional B Cells

Whereas B cells display anti-tumor activity, mainly sustained by their antibody production function, B cells can also be a crucial mediator of tumor growth. Indeed, circulating immune complexes (CICs) are composed of antibodies bound to a soluble antigen, and these complexes are known to induce inflammation through their recognition by the receptor of the fragment crystallizable region (FcR) [22]. Lisa Coussens’ lab demonstrated in a model of epithelium carcinogenesis (K14-HPV16 mice) that CICs produced by B cells, induced chronic inflammation by the activation of myeloid cells *via* engagement of the FcR [22,23]. Furthermore, B cell antibody production in the tumor-draining lymph nodes of melanoma (B16F10) bearing mice promotes tumor growth, which can be dampened by macrophages [40]. In addition to antibody production, Ammirante et al. demonstrated that tumor-infiltrating mature B2 cells (CD19^+^ B220^+^ CD5^−^ CD11b^−^) produce lymphotoxin (LT) which is crucial for castration-resistant prostate cancer tumor development. Tumor implantation in a mice specifically deficient for LT in B cells is associated with a significant growth delay [27]. Moreover, tumor-associated CD19^+^ B cells expressing the activated signal transducer and activator of transcription 3 (STAT3) can produce vascular endothelial growth factor (VEGF) at the tumor site, which leads to an increased angiogenesis and supports tumor progression [41].

#### 2.2.2. Regulatory B Cells

Besides the above-mentioned pro-tumorigenic activities, the tumor-promoting ability of B cells is mainly mediated by diverse populations of B cells known as regulatory B cells or Bregs. These cells are functionally defined by their capacity to mediate and maintain immune tolerance. Conventionally, Bregs were defined as CD5^+^ CD24^hi^ CD27^+^ CD38^hi^ B cells [42], but in the last few years different types of Bregs have been depicted, arising from different B cell subpopulations. It is also suspected that virtually all B cell subtypes could acquire a regulatory activity upon appropriate stimulation. Furthermore, some of Bregs subpopulations differ between mice and humans, making their characterization even harder. Nevertheless, one of the main feature of Bregs is the production of suppressive cytokines such as IL-10, IL-35 and TGF-β [5,6,7]. Lindner and colleagues showed that a Breg subset (CD19^+^ CD38^+^ CD1d^+^ IgM^+^ CD147^+^ granzyme B-expressing B cells) suppresses CD4^+^ T cell proliferation and causes Foxp3 expression in Tregs through secretion of IL-10 and TGF-β. This phenomenon is happening in several types of tumors, in particular in breast, ovarian, colorectal, cervical and prostate carcinomas [29]. In a very recent study performed on acute myeloid leukemia (AML) patients, Bregs are defined as CD19^+^ CD24^+^ CD38^+^. In this work, the authors show that AML patients display a higher frequency of Bregs and the presence of these cells predicts a short survival and poor prognosis [43]. Importantly, among the different phenotypic markers associated with mouse Bregs, immune checkpoints such as PD-1 and PD-L1 were recently pointed out. Xiao et al., described a Breg subset expressing high levels of PD-1 (PD-1^hi^ Bregs) in human hepatocellular carcinoma (HCC) samples. They showed how this Breg subset is able to suppress T-cell specific antitumor response and to promote tumor development through IL-10 signals [44]. Studying a mouse model of HCC, Shalapour et al. showed that class-switched IL-10-producing B cells are able to inhibit the anti-tumor response mediated by cytotoxic T cells through the interaction between PD-L1 expressed by Bregs and PD-1 expressed by T cells [45]. Olkhanud et al. define a previously undescribed subpopulation of “tumor-evoked” CD19^+^ B220^+^ CD25^+^ B cells (named tBregs) that promotes the development of tumor metastasis in the lungs of a breast cancer mouse model (4T1) [28]. A subpopulation of IL-10-producing CD19^+^ CD21^+^ Bregs able to suppress CD8^+^ IFN-γ^+^ T cells was also described in a murine model of skin carcinoma, and the differentiation of these cells is TNF-α–dependent [46]. A recent work by Das and Bar-Sagi demonstrated that Bruton’s tyrosine kinase (BTK) is able to promote Bregs differentiation, hence driving pancreatic carcinogenesis [47].

As already mentioned, one of the main feature of Bregs is the production of suppressive cytokines such as IL-10, IL-35 and TGF-β, and/or high level of expression of negative immune checkpoint molecules such as PD-L1. There are also several mechanisms used by Bregs to inhibit other immune cells, however their functional characterization is limited in the context of cancer. Shao et al. published an interesting work about the role of Bregs in directly promoting HCC development. Through in vivo and in vitro experiments, they observed that Bregs induce HCC cells proliferation, protect them from apoptosis and increase their migration capacities via the CD40/CD154 pathway [48]. Signals leading to differentiation of Bregs are multiple and not fully understood; however, examples of those signals can be find in the next sections.

## 3. Tumor Microenvironment Factors Influencing B Cells Functions

**Figure 2:**
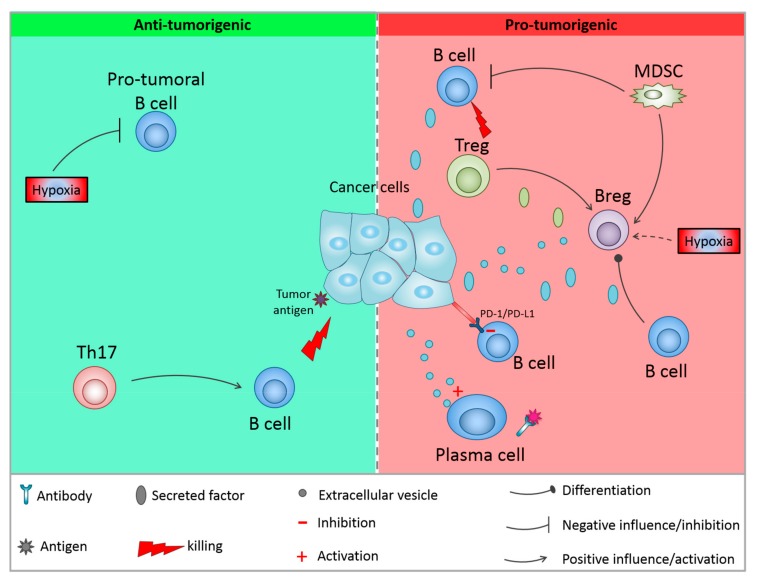
Influence of the microenvironment on B cells functions. Anti-tumor activities can be reinforced by different factors such as hypoxia or IL-17 production by T cells. On the other hand, other immune cells from the microenvironment can activate Bregs and kill anti-tumor B cells. In addition, the tumor cells themselves can influence B cells activity. CTL = cytotoxic T cells; Th17 = IL-17 producing T helper cell; MDSC = myeloid derived suppressive cell.

As discussed in the previous section, different B cell subpopulations can behave differently and have opposing functions. This can explain the dual role of B cells in both promoting cancer progression and anti-tumor immunity. In this section we will discuss the different factors of the TME that can influence these functions (Figure 2). The TME is highly immunosuppressive and a multitude of factors contribute to this status that can be sustained by cell-cell contacts, cytokine production or metabolic factors.

### 3.1. Influence of Different TME Cells on B Cell Functions

#### 3.1.1. Immune Cells Component Influencing B Cells

Tregs, which represent major players in tumor evasion, can directly influence B cells. A study performed by Lindner et al. showed that IL-21-producing Tregs found in human breast, ovarian, cervical, colorectal, and prostate carcinomas, induce CD19^+^ B cells differentiation into Bregs (CD19^+^ CD38^+^ CD1d^+^ IgM^+^ CD147^+^) expressing IL‑10, indoleamine 2,3-dioxygenase 1 (IDO1), and granzyme B (GrB). The transfer of active GrB to T cells promotes the degradation of the T-cell receptor (TCR), which blocks T cell proliferation [29]. In addition, the in vitro study performed by Zhao et al. described how CD4^+^ CD25^+^ Tregs, once activated, are able to suppress B cells proliferation by inducing granzyme-dependent cell death, affecting preferentially antigen–pulsed B cells (with APC capacities) [49]. These studies highlight the different behavior of Tregs in promoting B cells pro-tumoral activities by different means. Besides Tregs, other cell types support immune evasion such as myeloid-derived suppressor cells (MDSCs). In a model of breast cancer (4T1–bearing mice), it has been shown that these cells can induce the emergence of a Breg subset expressing PD-L1 but not PD‑1 [50]. In addition, MDSCs cells can also impair B cells function through IL-7 secretion which is associated with a decreased antibody production [51]. Notably, different cell types of the TME (T cells, DC, stromal cells, myeloid cells, NK cells) are able to secrete specific cytokines that can target B cells and potentially modulate their activity [52]. On the other hand, the cells found in the TME can also promote the anti-tumor functions of B cells. Interestingly, it has been described that tumor-infiltrating T cells expressing IL-17 can induce B cells migration and increase FAS/FASL–dependent direct tumor cell killing by B cells [36].

#### 3.1.2. Direct Action of Tumor Cells on B Cells

Tumor cells themselves can produce factors, cytokines or metabolites which directly influence B cells. Wejksza and colleagues, demonstrated that metabolites of the arachidonate 5-lipoxygenase (ALOX5) enzyme such as leukotriene B4 (LTB4) can activate the peroxisome proliferator–activated receptor α (PPARα) in B cells, inducing their differentiation in Bregs [53]. Furthermore, human breast cancer cells MDA-MB-231 express high level of chemokine (C-X-C motif) ligand 13 (CXCL13) which enable the migration of C-X-C chemokine receptor type 5 (CXCR5)–expressing B cells to the tumor [54]. Ricciardi et al. showed that co-culture of B cells with the cancer cells MCF7 induces apoptosis of B cells and appearance of a Breg population (CD56^+^ CD24^hi^ CD34^hi^) only when MCF7 underwent epithelial-to-mesenchymal transition (EMT), a process involved in cancer progression and metastasis and involving broad transcriptional and secretome changes [55]. Regarding melanoma cells, it has been demonstrated that they produce basic fibroblast growth factor (FGF2) which triggers the production of the tumor-supportive insulin-like growth factor 1 (IGF-1) by B cells present in the tumor niche [56]. Besides cytokines or soluble factor secretion, tumor cells communicate with their microenvironment through the secretion of extracellular vesicles (EVs), small structures which allow the trafficking of mRNAs or proteins. EVs derived from several tumor cell lines promote the expansion of a population of regulatory B cells expressing T-cell immunoglobulin and mucin-domain containing-1 (TIM-1) through a Toll-like receptor (TLR) dependent mechanism [57]. In addition, glioblastoma cells produce EVs carrying placenta growth factor (PIGF), which can convert naïve CD19^+^ B cells into regulatory B cells expressing TGFβ. This effect is antigen dependent as it is restricted to B cells infiltrating the tumor and not to peripheral B cells [58]. In vivo, tumor derived-EVs can induce increased antibody production by CD19^−^ CD138^+^ plasma cells in the tumor draining lymph node, which promote tumor growth [40].

### 3.2. Immune Checkpoint Stimulation on B Cells

One mechanism for tumor-induced immunosuppression is based on the expression of immune checkpoint molecules acting as receptor/ligand, including PD-L1/PD-1, galectin-9/TIM-3, IDO1, lymphocyte-activation gene 3 (LAG-3), and cytotoxic T-lymphocyte-associated protein 4 (CTLA4), which can inhibit the activation of effector lymphocytes. It is well described that PD-L1 is abnormally highly expressed on tumor cells [59]. On the other hand, PD-1 is expressed on a wide range of immune cells, including B cells [60]. When binding to its ligand, PD-1 can activate intracellular signaling pathways and inhibit the activation of B cells [61]. Okazaki et al. proposed a model explaining the PD-1-mediated B cell inhibition. They used a chimeric molecule composed of the extracellular region of the IgG Fc IIB receptor and the intracellular region of PD-1. They found that PD-1 prevented B cell receptor (BCR) signaling transduction through the recruitment and phosphorylation of tyrosine-protein phosphatase non-receptor type 11 (PTPN11). Once activated, PTPN11 dephosphorylates BCR-signaling molecules such as spleen tyrosine kinase (SYK), and impairs the downstream cascade, causing decrease in activation of phosphoinositide 3-kinase (PI3K), phosphoinositide-specific phospholipase C γ2 (PLCγ2), and extracellular signal–regulated kinases (ERK). This process impacts on calcium release and signaling [62]. In accordance with this work, Haas and colleagues showed that PD-1 interaction with PD-L1 suppresses antigen-specific proliferation of B1-b cells (B220^lo^ CD19^hi^ CD1d^int^ CD21/35^lo^ CD11b^+^ CD5^neg-lo^) and their secretion of antibodies during T cell independent activation [63]. Moreover, PD-1 prevents the production of antibodies by B cells in a model of immunization against the Thomsen-nouvelle (Tn) antigen in tumor-bearing mice [64].

### 3.3. Effect of Hypoxia on B Cells

One hallmark of cancer is hypoxia, which is a major difference between tumor and normal tissues [65]. At the tumor site, the cell number increases, immune cells infiltrate the tumor and a general vascular disorganization occurs. All these mechanisms cause an increased oxygen consumption and decreased oxygen supply, leading to a hypoxic condition which affects both the tumor cells and the tumor-infiltrating immune cells. Hypoxia-inducible factors (HIFs) regulate a transcriptional network crucial for the adaptation of cells to this environment, involving metabolism adaptation from oxidative phosphorylation to glycolysis. In tumor cells, HIF induces the secretion of factors that can regulate immune cell recruitment to support tumor growth [66]. In addition, hypoxia and the HIF signaling pathway can modulate metabolism and impact on a wide variety of cellular processes in immune cells [67,68]. The influence of these mechanisms on B cells is poorly elucidated in the context of cancer, however several lines of evidence suggest that hypoxia influences B cells functions. B cell-specific deletion of glucose transporter 1 (Glut1), a HIF-1α target gene, leads to a decrease in B cells proliferation and antibody production capacities [69]. TWIK–related acid–sensitive K^+^ channel 2 (TASK-2) is a potassium channel, the expression of which is controlled by HIF-1α in B cells under hypoxic conditions. TASK-2 hypoxia-dependent expression is correlated with elevated calcium flux, suggesting that this protein could be crucial for B cells functions [70]. An interesting study performed by Meng and colleagues demonstrated that HIF-1α contributes to IL-10 production by CD1d^hi^ CD5^+^ B cells, as it directly regulates IL-10 expression during autoimmune disease [71]. As described previously, IL-10 is one of the key immunosuppressive cytokines released by Bregs within the TME. Hence, the same mechanism may occur in the tumor context, where sustained hypoxia may support the immunosuppressive functions of regulatory B and T lymphocytes. In the context of cancer, it has been reported that HIF-1α stabilization under hypoxic conditions prevents CD19^+^ B cells from colonizing the tumor site and is associated with a slower development of the tumor in the early stage of pancreatic cancer development [72].

## 4. Role of B Cells in Cancer Therapy

**Figure 3:**
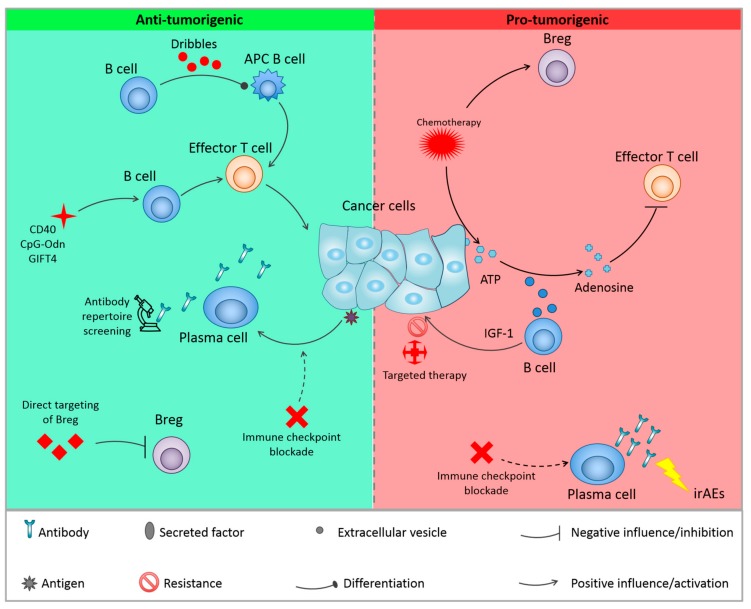
Cancer therapies and B cells. Current therapies can influence B cells functions, leading to resistance to therapy by the activation of Breg, or release of ATP which is converted into adenosine by B cells Extracellular vesicles, leading to the inhibition of T cells. Activation of antibody production is associated with adverse side effects in immunotherapy. On the other hand, other therapies activate anti-tumoral activities of B cells either by activating B cells with anti-tumorigenic functions or by inhibitions of Bregs. APC = antigen presenting cell; irAEs: immune-related adverse effects; IGF-1: insulin-like growth factor 1.

Considering the role of B cells in tumor development, it is not surprising that B cells could also have an impact on cancer therapy. It has been described that B cells could help to predict response to some therapies, or immune-related adverse effect (irAE). In addition, B cells can directly impact the efficiency of some cancer treatments (Figure 3, Table 2). Efforts have been made in order to target these cells, either through their activation or, on the contrary, through their depletion/inhibition (Figure 3, Table 3).

### 4.1. B Cells in Therapy: Implication in Resistance and Correlation with Response

In addition to their direct effect on tumor cells, chemotherapies induce an immune response, which is triggered by the induction of immunogenic cell death [73]. The tumor immuno-suppressive microenvironment can then impede the efficiency of these therapies. Oxaliplatin treatment was reported to be associated with an increased infiltration of immunosuppressive B cells expressing IgA, IL-10 and PD-L1 in the transgenic adenocarcinoma of the mouse prostate (TRAMP) model of metastatic prostate cancer (PC). In Human, these cells are also found enriched in therapy resistant patients [74]. Squamous cell carcinomas (SCC) are also highly enriched in CD20^+^ B cells, which are at least in part responsible for the resistance to platinum- (cisplatin and carboplatin) and taxol-based (paclitaxel) chemotherapy as their removal using an anti-CD20 antibody prevents this resistance [75]. Very recently, Zhang et al. elegantly demonstrated that chemotherapy-induced ATP released from damaged tumor cells is hydrolysed into adenosine by B cells-derived EVs in the TME. This elevated rate of adenosine inhibits the activation of effector T cells, preventing efficient response to chemotherapy in mice model of melanoma (B16F10) and colon adenocarcinoma (MC38). In a cohort of colon cancer patients, the authors observed that these CD19+ EVs are associated with bad prognosis and suggested that they could be used as a biomarker for chemotherapy resistance [76]. As opposed to chemotherapy, targeted therapy involves the modulation of specific pathways involved in oncogenic processes, such as signaling kinases. In melanoma, v-Raf murine sarcoma viral oncogene homolog B (BRAF) and MAPK (mitogen-activated protein kinase)/ERK kinase (MEK) inhibitors are widely used. However, despite high level of initial response rate, a high percentage of patients will develop resistance [77]. Somasundaram and colleagues demonstrated that tumor-associated B cells induce resistance to these inhibitors in vitro through the secretion of IGF-1 and confirmed an increase of CD20 and IGF-1 gene expression in tumor of resistant patients [56].

Immunotherapy constitutes the reactivation of the immune system which is mainly immunosuppressive in the TME [78]. Despite the fact that immunotherapy has revolutionized the field of oncology in the last few years, a high proportion of patients still don’t benefit from this advance. Majority of these therapies are designed to target effector T cells. However, it is increasingly clear that B cells can also be affected by these treatments, and that these cells play a role in the response to these therapies. Indeed, pre-existing antibodies against the tumor antigen NY-ESO-1 in the serum of patients with melanoma correlate with an improved clinical benefit following anti-CTLA4 immunotherapy [79]. In the blood of non-progressing patients with different forms of metastatic cancers (melanoma, lung adenocarcinoma, non-small cell lung carcinoma and renal cell carcinoma) treated with immune checkpoint blockade immunotherapy (anti-CTLA4, anti-PD-1) or by chemotherapy, an active humoral immune response could be detected, characterized by somatic hypermutation, IgG class switch and clonal expansion of plasmablast (CD20^−^ CD19^+^ CD38^hi^ CD27^+^ CD3^−^ CD14^−^ IgA^−^ IgM^−^). Interestingly, the antibodies produced in these patients recognized antigens found in several types of cancer and across patients [80]. In 2018, Sade-Feldman et al. analyzed at single-cell level the immune microenvironment of melanoma patients responding, or not responding, to immunotherapy by the use of cutting-edge technologies (mass cytometry and single-cell RNA sequencing). They observed an enrichment of B cells in responder patients [81]. These reports suggest a beneficial role of B cell activation for immunotherapy efficiency. However, expression of immune checkpoints is not restricted to T cells, and these molecules are also found at the surface of B cells, like PD-1 [82], CTLA4 [83], or LAG-3 [84,85], implying that the immune checkpoint blockade could directly affect B lymphocytes activity.

The interruption of treatment due to side effect is a major pitfall of immunotherapy. These side effects are known as immune-related adverse effects (irAEs) and they are due to the overactivation of the immune system which can then attack different organs [86]. The prediction of patients who will develop irAEs, and the understanding of the mechanisms involved are viewed as main challenges to bring forward this new class of treatment. Das et al. observed a decrease in the total number of B cells in the blood of melanoma patients treated with combination checkpoint blockade (CCB) therapy (anti-CTLA4 and anti-PD-1 antibodies). However, CD21^lo^ B cells expressing a high level of PD-1 were, in contrast, enriched and activated, and patients with this specific B-cell response have a higher risk to develop irAEs [87]. In melanoma patients treated with bacillus Calmette-Guerin (BCG) followed by CTLA4 blockade, increase in tumor autoantibody repertoire precedes high grade irAEs [88,89]. These results highlight the potential roles played by B cells in immunotherapy side effects, and their potential role as biomarkers. 

### 4.2. Therapies Activating B Cells

The activation of B cells appears to be a promising approach in the fight against cancer, and several strategies have been developed in order to fully unleash the anti-tumor potential of B cells. B cell–based cancer vaccines consist in the stimulation of B cells in order to activate cytotoxic T cells against tumors. In this context, the use of CD40 stimulation has been widely studied. Indeed, the ligation of CD40 with its ligand CD40L induces the expression of co-stimulatory molecules and cytokines, and CD40-activated B cells gain the potential to promote the activation of naïve and memory T cells [90,91]. In addition, it has been shown that these CD40-activated B cells are not sensitive to the immunosuppressive microenvironment [92], and that they can efficiently reach secondary lymphoid organs when injected in vivo, where they can efficiently activate T cells [93]. The potential of these CD40-activated B cells have been tested and validated in vivo in models of Human papillomavirus 16 (HPV16) E6 and E7 expressing TC-1 tumor [94], B16-F10 melanoma, E.G7 lymphoma [95], 4T1 breast tumor metastasis [96], sarcoma [25] and in spontaneous non-Hodgkin’s lymphoma in dogs [97]. In conclusion, CD40-activated B cells represent an interesting tool in cancer immunotherapy, and further studies, including clinical trials, should be performed to confirm this potential. Cytosine guanine dinucleotide-oligodeoxynucleotides (CpG-ODN), is a Toll-like receptor 9 (TLR9) ligand that can also be used to activate B cells. In a model of B16-F10 derived lung metastases, injection of CpG-activated B cells induces a regression of metastases and a less immunosuppressive microenvironment [98]. The “fusiokine” GIFT4, is a fusion between GM-CSF and IL-4 cytokines which unexpectedly cluster the respective receptors on B cells and leads to the activation of Janus kinase (JAK)/STAT pathway in these cells. This clustering triggers the proliferation of B cells and their differentiation from naïve B cells to activated helper B cells up-regulating CD19, CD25, CD27, CD40, CD69, MHC class I and II, CD80, CD83 and CD86 expression. These activated B cells act as APCs, secrete cytokines and express co-stimulatory markers, leading to the activation of T cells into cytotoxic T cells. Treatment of mice bearing melanoma with GIFT4 leads to an efficient control of tumor growth. This effect is B cell-dependent as tumors are resistant to GIFT4 in B cell-deficient mice [99]. Otherwise, tumor-derived autophagosomes enriched in defective ribosomal products (DRibbles) can be captured and internalized by B cells. These DRibbles contain tumor specific antigens, and lead to the activation of B cells associated with increased expression of MHC class I and II molecules, CD86 and CD40. These cells can then present DRibbles-derived antigens to stimulate tumor specific T cell response. The combined injection of DRibbles and DRibble-loaded B cells in mice bearing lymphoma and hepatocellular carcinoma induce the control of tumor growth [100,101].

Antibodies produced by B cells are associated with therapeutic efficiency. Several examples of antibody-based therapies can be cited, with various clinical outcome, some of them targeting directly tumor antigens (anti-CD20 in B-cell related lymphoma/leukemia, anti-HER2 in breast cancer), others having immunomodulatory effects such as the one used for immune checkpoint blockade (anti-CTLA4, anti-PD-1/PDL-1, anti-LAG) (reviews on the subject can be found in [102,103,104]). Indeed, antibodies have the capacity to activate antibody-dependent cellular cytotoxicity, phagocytosis, and complement-dependent cytotoxicity and can modulate signaling in target cells by activation or inhibition of cell surface receptors [102]. The antibodies produced by B cells found in the tumor and in tumor-draining lymph nodes have in theory the capacity to induce these effects but are certainly produced at too low a concentration to be effective. One strategy under investigation to increase the antibody repertoire available in the clinic aims at identifying tumor–reactive antibodies by the characterization of antibody repertoire of these tumor–infiltrating B cells by screening a phage–display library [105,106] or by next–generation sequencing (NGS) [107,108,109].

### 4.3. Therapies Depleting/Inhibiting B Cells

As some B cell populations are associated with pro-tumoral activities, the idea of depleting or inhibiting B cells has also been explored by the scientific community. Anti-CD20 antibodies were initially design for the treatment of autoimmune diseases and B cell lymphomas. However, it became clear that these antibodies could be used to deplete B cells in solid tumors. Depletion of B cells using an anti-CD20 antibody in small cohorts of patients with advanced colon cancer [110], melanoma [111,112], cutaneous T cell lymphoma [113] was associated with health benefit. However, it can also be deleterious in another context. For example, it leads to an increased tumor growth in B16-F10 melanoma bearing mice [114] and in the lung of mice intravenously injected with melanoma B16-F10 cells [98]. In addition, a study reported no clinical benefit in patients with renal cell carcinoma and melanoma treated with anti-CD20 antibody [115]. These contradictory results could be explained by the cell populations targeted by the anti-CD20 antibody. Indeed, it has been reported that these antibodies could efficiently cleared B cells expressing high level of CD20, without affecting cells with a low level of expression, which leads to an enrichment in regulatory B cells and to an even more immunosuppressive environment [116].

It is worth noting that Bruton’s tyrosine kinase (BTK) inhibitor ibrutinib used for the treatment of some B cell-associated lymphomas/leukemias has been tested in solid tumors [117]. Indeed, BTK activity is not restricted to B cells, but is also involved in oncogenic pathways in solid tumors, and is activated in myeloid cells, including MDSCs [118]. However, it is clear that BTK activity in these solid tumors could also be at least partially explained by its direct activity on B cells. In that respect, in a mouse model of pancreatic ductal adenocarcinoma, treatment with BTK inhibitor induces a reduction in tumor growth which was associated with a depletion or decreased presence of IgM^lo^CD23^+^CD5^−^ follicular and IgM^lo^ CD23^−^ memory B cells [119].

Specific inhibition of Bregs is perceived as a sensitive way to divert the immunosuppressive TME, without affecting the B cells populations that could be, on the contrary, beneficial for anti-tumor immunity. The Biragyn’s lab managed to efficiently deplete CXCR5+ Bregs in vivo by coupling CXCL13 (CXCR5 ligand) to CpG-ODN. After injection in tumor-bearing mice, the number of Bregs was reduced, and in contrast, remaining B cells had the capacity to induce activation of cytotoxic T cells [116]. Another strategy described is the specific inhibition of Bregs with a blocking antibody against IL-10 which leads to an increase efficiency of CD40–activated B cells transfer [6]. Some molecules have also been associated with inhibition of Bregs cells. The phytoalexin resveratrol, which is known to inhibit STAT3 phosphorylation, induces a decreased proportion of Bregs in breast tumor (4T1)–bearing mice and blocks lung metastases formation [120]. It is known that the components of the total glucosides of paeony (TGP), extracted from plant, are important for their anti-inflammatory and immunomodulatory activities. In a model of diethylnitrosamine (DEN)-induced hepatocellular carcinoma in rats, TGP treatment leads to a reduction of nodules and improvement of survival through a reduction of Breg numbers [121]. Lipoxin A4 (LXA4), a metabolite of the arachidonic acid pathway, inhibits B16F10 melanoma and H22 hepatocarcinoma tumor growth which could be at least partially explained by a decrease in Bregs. In vitro, LXA4 prevents differentiation in Bregs but also Breg-induced Tregs differentiation [122]. Interestingly, another metabolite from this pathway has the opposite effect: the LTB4 is able to induce conversion of naïve B cells into Bregs. MK886, an inhibitor of ALOX5 enzyme, required for LTB4 production, leads to reduced differentiation capacities of B cells into Bregs and control the growth of B16F10 tumors. Transfer of Bregs restores the tumor growth, whereas MK886-treated ones fail to do so [53]. All these molecules represent interesting potential therapeutic approaches in cancer, however their efficiency and mechanism of action need to be attested into more details and in a higher variety of models. 

## 5. Conclusions and Perspectives

B cells tend to be overlooked for their role in the anti–tumor immunity. Nevertheless, they represent fundamental players in the TME, where they can either enhance an efficient immune response by activating cytotoxic T cell response, producing anti-tumor antibodies and cytokines, or inhibit immunity and participating in cancer immune evasion. These contradictory activities are achieved by different B cell populations, which can be induced or on the contrary inhibit in the TME. Interestingly, they are also involved in the response to therapy. In this context, efforts should be done in order to either activate B cells with anti-tumor activities or inhibit the regulatory B cells. In fact, a better understanding of the B cell sub-populations appears to be essential in order to be able to develop new strategies to target them specifically. The identification of specific signaling pathways, expression of cell surface markers and immune checkpoint molecules, or dependency to cytokines could serve as targets for the development of these therapies. In addition, limiting identification of B cell to a single marker (generally CD20 or CD19) when studying prognosis/predictive value of B cells appears to restrict the comprehensive utility of such studies. In this context it is clear that more markers should be included in order to distinguish different B cells populations. In the last few years, with the development of cutting-edge technologies such as mass cytometry or single-cell RNA-sequencing, the fine analysis of immune sub-populations has become possible. In the context of TME, dozens of publications analyzed immune infiltration using these technologies. Unfortunately, the majority of the analyses investigated T or myeloid cells, with very little focus on the B cell populations, even in the case where they represented an important fractions of the immune cells [12,13,14,15,16,17,18,81]. The use of these powerful tools should be applied to B lymphocytes, as they remain mysteries that need to be solved in order to fully develop their potential in the fight against cancer.

## Figures and Tables

**Table 1:** Overview of the molecules produced by B cells in the context of cancer and their effect on tumor immunity.

Effector Molecules/Mechanism	Function		Reference
	Pro-Tumorigenic	Anti-Tumorigenic	
**Antibodies**	Circulating immune complexes activate Fcγ receptors on immunosupressive myeloid cells, facilitate angiogenesis	Antibodies against tumor antigens, mediate complement -dependent lysis, ADCC, FcR-mediated phagocytosis, antigen presentation by DCs	[22,23,24,25]
**Fas/FasL**	Bregs inducing apoptosis in CD4^+^ T cells	Killing of tumor cells	[6]
**TRAIL/Apo2L**		Killing of tumor cells	[26]
**IL-10**	Produced by Bregs, exacerbate inflammation and support cancer growth, inhibit CD4^+^ T cells		[5,6,7,22,23,27]
**TGF-β**	Convert naïve CD4^+^ T cells into Foxp3^+^ Tregs, upregulate reactive oxygen species (ROS) and nitric oxide (NO) in MDSCs		[28]
**Granzyme B**	Transfer to T cells, degrading the T cell receptor ξ chain without inducing T cell apoptosis	Induce apoptosis in B-chronic lymphocytic leukemia cells	[29,30]
**Lymphotoxin**	Activates non-canonical and canonical NF-κB signaling and STAT3, inhibitory effect of B cells, survival signals to tumor cells		[27]
**IL-35**	Stimulates tumor growth		[31]
**IFN-y**		Facilitate the killing of tumor cells by NK cells, polarize T cells towards Th1 or Th2 response	[5,6,7]

**Table 2:** The roles of B cells in response to current therapies are summarized accordingly to the type of therapy and B cell subsets.

Effect	Type of Therapy	B Cell Subtype/Function	Cancer Type/Mouse Model/Cell Line	References
Resistance	Oxaliplatin	IgA^+^ IL10^+^ PD-L1^+^	Prostate	[74]
	Platinum/Taxol-based chemotherapy	CD20^+^	Carcinoma	[75]
	Chemotherapy (oxaliplatin, doxorubicin, phosphoramide mustard, cyclophosphamide	CD20^+^ EVs	B16F10, MC38	[76]
	BRAF/MEK inhibitor	IGF-1 producing CD20^+^	B16F10	[56]
Positive impact on response	Anti-CTLA4	Presence of tumor specific antibodies	Melanoma	[79]
	Anti-CTLA4, anti-PD-1, chemotherapy	Somatic hypermutation, IgG class switch, clonal expansion of B cells	Melanoma, lung and renal cell carcinomas	[80]
	immunotherapy	CD20^+^ enrichment	Melanoma	[81]
Prediction of irAEs	Anti-CTLA4 and anti-PD-1	CD21^lo^ PD-1^hi^ enrichment	Melanoma	[87]
	BCG + anti-CTLA4	Increase in anti–tumor antibodies repertoire	Melanoma	[88,89]

**Table 3:** The different anti-cancer therapies targeting B cells, either through their activation or on the contrary through their depletion/inhibition are summarized.

Type of Therapy	Target Cells/Effect on B Cells	Consequence	Cancer Type/Animal Model/Cell Line	References
Activation of B cells				
B cell–based vaccine–CD40 dependent activation	CD19^+^	Activation of T cells, migration to secondary lymphoid organs	HPV16, B16-F10, E.G7, 4T1 metastasis, sarcoma, spontaneous NHL.	[25,92,94,95,96,97]
CpG-ODN	CD19^+^	Metastasis regression, decrease immunosuppressive TME	B16-F10	[98]
GIFT4	Up-regulation of CD25, CD27, CD40, CD69, MHC class I/II, CD80, CD83 and CD86 expression	Activation of CTL	B16-F10	[99]
DRibbles	MHC class I and II molecules, CD86 and CD40	Activation of tumor specific T cells	Lymphoma, HCC	[100,101]
Inhibition/Depletion of B cells				
Anti-CD20 antibody	Depletion of CD20^+^ cells	Health benefit	colon cancer, melanoma, cutaneous T cell lymphoma	[110,111,112,113]
		No effect or deleterious effect	B16-F10 Renal cell carcinoma, melanoma	[98,114,115,116]
BTK inhibitor	Depletion of IgM^lo^ CD23^+^ CD5^−^ and IgM^lo^ CD23^−^ B cells	Reduction in tumor growth	Pancreatic ductal adenocarcinoma	[117]
CXCL13-CpG-ODN	Depletion of CXCR5+ Bregs	Activation of CTL	4T1	[116]
Anti-IL10 antibody	Inhibition of Bregs	Increase efficiency of CD40-activated B cells	4T1	[6]
Resveratrol	Decrease in Breg number	Block metastasis formation	4T1	[120]
Total glucosides of paeony (TGP)	Decrease in Breg number	Improved survival	HCC	[121]
Lipoxin A4	Inhibition of Breg conversion from naïve B cells	Decrease tumor growth	B16-F10, H22	[122]
MK886	Inhibition of Breg conversion from naïve B cells	Decrease tumor growth	B16-F10	[53]
